# Publication trends of research on COVID-19 and host immune response: A bibliometric analysis

**DOI:** 10.3389/fpubh.2022.939053

**Published:** 2022-08-08

**Authors:** Yun Xia, Ren-qi Yao, Peng-yue Zhao, Zheng-bo Tao, Li-yu Zheng, Hui-ting Zhou, Yong-ming Yao, Xue-min Song

**Affiliations:** ^1^Department of Anesthesiology, Zhongnan Hospital of Wuhan University, Wuhan, China; ^2^Translational Medicine Research Center, Medical Innovation Research Division and Fourth Medical Center of the Chinese People's Liberation Army General Hospital, Beijing, China; ^3^Department of Burn Surgery, The First Affiliated Hospital of Naval Medical University, Shanghai, China; ^4^Department of General Surgery, First Medical Center of the Chinese People's Liberation Army General Hospital, Beijing, China; ^5^Department of Orthopedics, Changzheng Hospital, Naval Medical University, Shanghai, China; ^6^Institute of Pediatric Research, Children's Hospital of Soochow University, Suzhou, China

**Keywords:** SARS-CoV-2, COVID-19, immune response, sepsis, bibliometric analysis

## Abstract

**Introduction:**

As the first bibliometric analysis of COVID-19 and immune responses, this study will provide a comprehensive overview of the latest research advances. We attempt to summarize the scientific productivity and cooperation across countries and institutions using the bibliometric methodology. Meanwhile, using clustering analysis of keywords, we revealed the evolution of research hotspots and predicted future research focuses, thereby providing valuable information for the follow-up studies.

**Methods:**

We selected publications on COVID-19 and immune response using our pre-designed search strategy. Web of Science was applied to screen the eligible publications for subsequent bibliometric analyses. GraphPad Prism 8.0, VOSviewer, and CiteSpace were applied to analyze the research trends and compared the contributions of countries, authors, institutions, and journals to the global publications in this field.

**Results:**

We identified 2,200 publications on COVID-19 and immune response published between December 1, 2019, and April 25, 2022, with a total of 3,154 citations. The United States (611), China (353), and Germany (209) ranked the top three in terms of the number of publications, accounting for 53.3% of the total articles. Among the top 15 institutions publishing articles in this area, four were from France, four were from the United States, and three were from China. The journal *Frontiers in Immunology* published the most articles (178) related to COVID-19 and immune response. Alessandro Sette (31 publications) from the United States were the most productive and influential scholar in this field, whose publications with the most citation frequency (3,633). Furthermore, the development and evaluation of vaccines might become a hotspot in relevant scope.

**Conclusions:**

The United States makes the most indispensable contribution in this field in terms of publication numbers, total citations, and H-index. Although publications from China also take the lead regarding quality and quantity, their international cooperation and preclinical research need to be further strengthened. Regarding the citation frequency and the total number of published articles, the latest research progress might be tracked in the top-ranking journals in this field. By analyzing the chronological order of the appearance of retrieved keywords, we speculated that vaccine-related research might be the novel focus in this field.

## Introduction

Coronavirus disease 2019 (COVID-19), caused by severe acute respiratory syndrome coronavirus 2 (SARS-CoV-2), is currently in global pandemic since its first emergence in late December 2019. Following the statistic from the World Health Organization (WHO), as of April 25, 2022, the cumulative number of confirmed COVID-19 cases exceeds 511 million with more than 6 million deaths (1). Even more, a study has revealed that more than half of hospitalized patients who recovered from the COVID-19 episode have experienced residual multisystem symptoms that lasted for 6–12 months (2), namely, post-COVID-19 condition (3). Undoubtedly, a thorough yet comprehensive understanding of the pathogenesis of SARS-CoV-2 infection will facilitate the implementation of effective treatments.

The pathophysiological features of SARS-CoV-2 infection involve viral invasion, malfunction of the immune response, dysregulation of the renin-angiotensin-aldosterone system, endothelial cell injury, and microcirculation dysfunction (4), in which immune response is of prominent significance. Correspondingly, numerous studies have confirmed that SARS-CoV-2 poses great threats to host immune responses and causes COVID-19-associated immune dysfunction, which induces ARDS and multiple organ failures leading to severe and critical disease development (5). A significant pathophysiological feature of severe and critical COVID-19 cases is cytokine release syndrome (CRS) (6, 7), in which interleukin (IL)-1β, IL-6, IL-8, IL-10, IFN-γ, TNF-α, and various cytokines are released in large quantities by various immune and non-immune cells, thereby triggering potent cytokine storm and tissue damages (8–11). T cells exhaustion (12) and consequent lymphocytopenia are another hallmark of SARS-CoV-2 infection and are relevant to the worsening clinical prognosis and outcomes of COVID-19 patients (13). Additionally, a multicenter observational study has shown that COVID-19 and bacterial sepsis share the same biological host response regarding endothelial cell damage and microcirculation dysfunction (14). Specifically, the expression of pro-inflammatory mediators and microcirculation alterations is consistent between patients with COVID-19 and bacterial sepsis, which are closely related to the severity of the disease. Comprehending immune-related pathogenesis of SARS-CoV-2 infection and COVID-19-associated immune dysregulation will benefit global researchers in the development of novel monitoring indicators, as well as therapeutic targets (15–17), which can facilitate us in reducing fatality rates and improving clinical outcomes for COVID-19 patients.

The bibliometric analysis represents a methodology combining mathematics and statistics, which have long been applied to analyze the literature in quantitative and qualitative over time to depict and predict the publication trend in a certain research field (18, 19). Meanwhile, bibliometric analysis can provide detailed information concerning the contribution of disparate countries, scholars, and institutions to global publications, thereby mapping their connection and relationship to imply international cooperation. The integration and analysis of this information can facilitate global scholars in mastering the trendy topic and latest progress in a certain field. More importantly, bibliometrics can serve as a reliable guide for implementing clinical treatment, as well as formulating policy against various human diseases. Several bibliometric analyses published recently have covered multiple dimensions of COVID-19-related studies (20, 21), including coronavirus research (18) and clinical research (22). However, there is no study specifically addressing COVID-19 and immune response *via* bibliometric methodology.

Based on the Web of Science (WOS), the current study was carried out to analyze the status of research on COVID-19 and immune response using bibliometric analysis. As the first bibliometric analysis of COVID-19 and immune responses, this study will provide researchers with a comprehensive overview of the latest research advances. We attempt to summarize the scientific productivity and cooperation across countries and institutions using the bibliometric methodology. Meanwhile, using clustering analysis of keywords, we revealed the evolution of research hotspots and predicted future research focuses, thereby providing valuable information for the follow-up studies.

## Materials and methods

### Data sources and retrieval strategies

Web of Science (WOS) represents a comprehensive, multidisciplinary database that provides information related to references of all publications, authors, authors' affiliated institutions, publishers, etc. With the powerful function and citation reports, it can quickly target high-impact studies, find the research direction concerned by the authorities at home and abroad, and reveal the development trend of the subject. It has become the most adopted database for bibliometric analysis (18–20).

We chose Web of Science Core Collation (WoSCC) as our primary database for performing bibliometric analysis, for which we limited the type of publications to original articles and reviews in English. The searching timespan was from December 1, 2019, to April 25, 2022. All data were obtained and downloaded from a publicly available database and did not involve any ethical issues requiring approval.

**Figure 1:**
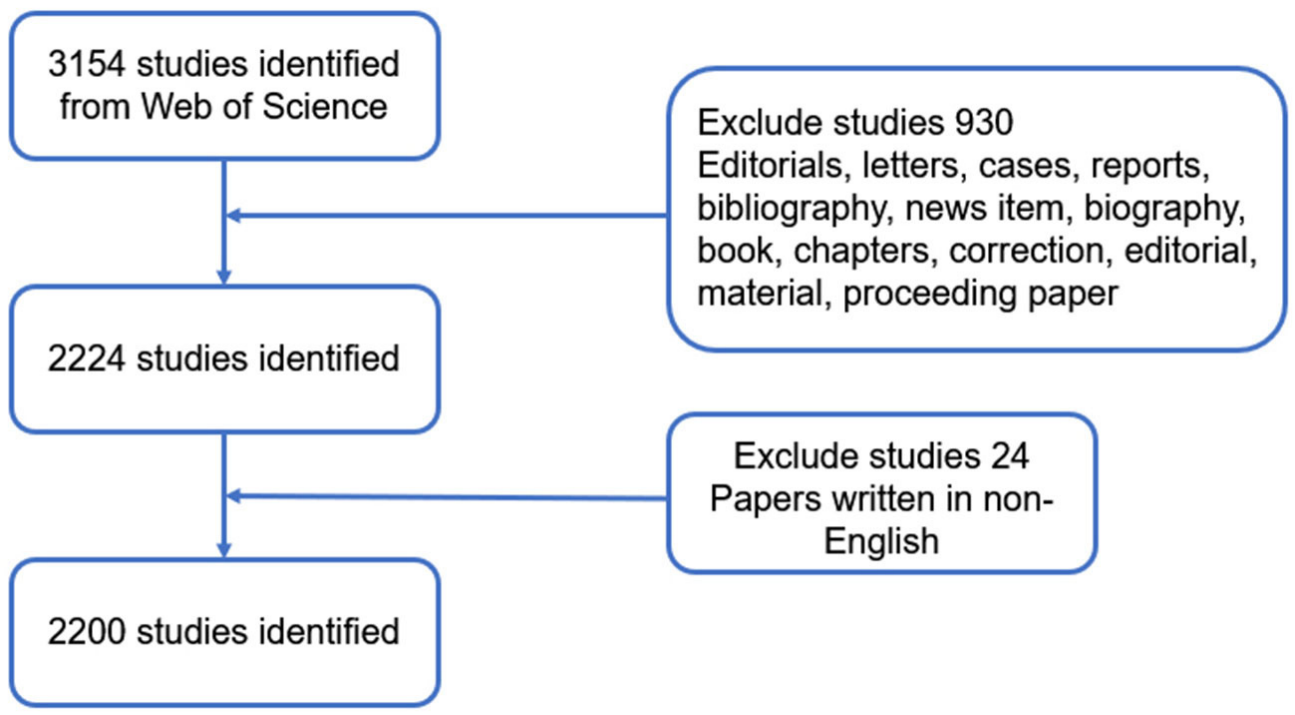
Detailed flowchart of search, screening, and registration on the Web of Science.

Due to the daily renewal of the database, data retrieval was carried out within a single day on April 25, 2022. The applied search strategy was as follows: TI = [macrophage OR neutrophil OR (NK cell) OR (natural killer cell) OR (myeloid derived suppressor cell) OR MDSC OR (innate lymphoid cell) OR ILC OR (dendritic cell) OR DC OR (T cell) OR (T lymphocyte) OR (B cell) OR (B lymphocyte) OR (plasma cell) OR (regulatory T cell) OR (Treg) OR (monocyte) OR (immunoglobulin) OR immunosuppression OR (immune dysfunction) OR (immune response)] AND TI = [(COVID 19) OR (2019 novel coronavirus) OR (coronavirus 2019) OR (coronavirus disease 2019) OR (2019-novel CoV) OR (2019 ncov) OR (COVID 2019) or COVID19 OR (corona virus 2019) OR (nCoV-2019) or nCoV2019 OR (nCoV 2019) OR (2019-ncov) or (COVID-19) OR (Severe acute respiratory syndrome coronavirus 2) OR (SARS-CoV-2)] AND LANGUAGE: (English) AND DOCUMENT TYPES: (Article OR Review). Detailed information regarding to literature screening were presented in Figure 1.

### Data collection

Two reviewers (YX and P-yZ) collected detailed information from all incorporated publications, respectively, including country/region, keywords, published journals, citations, H-index, and so on. Thereafter, the extracted data were processed and analyzed using Microsoft Excel 2016 (Redmond, Washington, USA), VOSviewer (Leiden University, Leiden, the Netherlands), and GraphPad Prism 8.0 (GraphPad Prism Software Inc., San Diego, CA). The figures of results were integrated by Adobe Illustrator CS6 (Adobe Systems Incorporated, SAN Jose, California, USA).

### Bibliometric analysis

The H-index means that a researcher has published H articles, and each of his articles has been cited at least H times. It is a well-established indicator to evaluate the quantity and quality of academic output from a certain scientific researcher or country/region. The impact factor (IF) was determined by inquiring about the latest version of Journal Citation Reports (JCR). Analysis of characteristics concerning published journals, authors, and countries/regions, the total number of citations, H-index, and a total number of publications were conducted by applying Web of Science. Moreover, GraphPad software was used to visualize the data. VOSviewer could automatically map and visualize the network of co-authorship, keywords co-occurrence, citation, bibliographic coupling, co-citation, and research hotspots. We chose full-counting as the counting method, and threshold settings for minimum numbers were described in detail in the results section. In the cluster analysis generated by VOSviewer, each dot represented a publication or keyword, and the size of each dot represented the frequency of co-occurrence. Lines connected relevant publications or keywords, the thickness of which indicated their linking strength. Moreover, VOSviewer could visualize the average appearing time of each keyword, which implied possible research trends in a certain area. In the clustering analysis of CiteSpace, the keyword co-occurrence network was composed of multiple clusters, and each one was assigned a label. The number of the label represented the size of the cluster. Co-citation referred to the relationship between two references that were simultaneously cited by other documents. The co-occurrence of keywords referred to the simultaneous occurrence of some keywords in all analyzed publications with a certain frequency.

## Results

### Contributions of countries/regions to global publications

Overall, a total of 2,200 publications retrieved from Web of Science met our inclusion criteria and were subjected to subsequent analyses. The total citation frequency of all included articles was 47,681, with an average citation per article of 21.67 and an H-index of 94.

**Figure 2:**
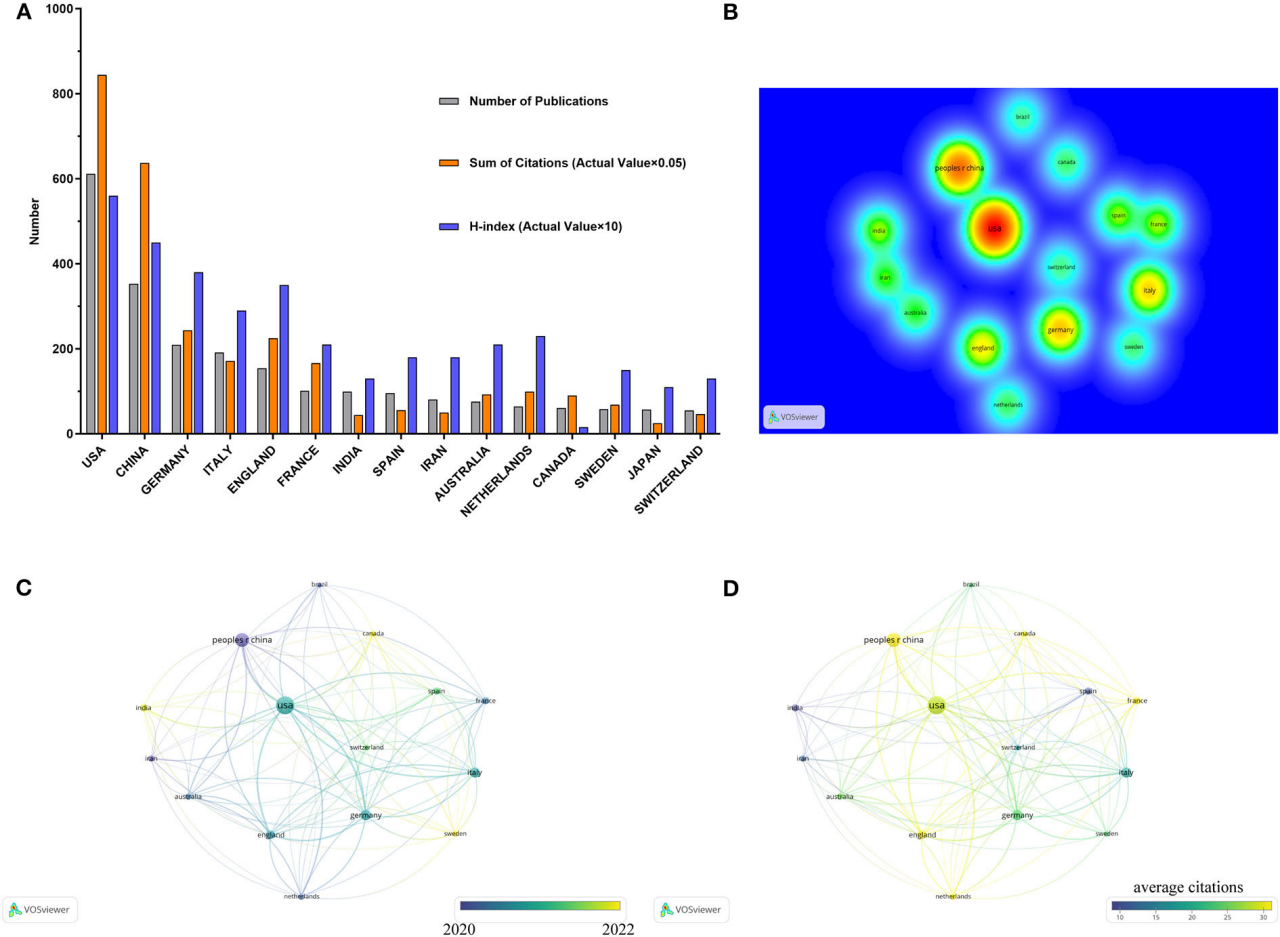
Publication of articles in various countries/regions. **(A)** The total number of citations (×0.05) and H-index (×10) of articles from the top 15 countries ranked by publications' number. **(B)** Density map of the top 15 countries ranked by number of publications. **(C)** International cooperation among top 15 countries. **(D)** The top 15 countries were shown in chronological order of publications.

Among all eligible articles, the United States (611, 27.8%), China (353, 16.0%), and Germany (209, 9.5%) ranked the top three countries in terms of publication numbers. Publications from the United States had 16,890 citations (15,752 times without self-citations), accounting for 35.4% of the total citations, with an H-index of 56. The citation frequency of China was 12,737 (26.7%, 12,359 times without self-citations), with an H-index of 45. Germany ranked third in terms of publication numbers, total citations, and H-index, followed by Italy, England, and France (Figure 2A).

In the thermodynamic chart of Figure 2B and the co-authorship network map of Figure 2C, the United States was highlighted, spatially distributed at a central position, and had relatively more connections to other countries/regions, indicating closer international cooperation compared to the others. Considering the publication time of literature on immune response and COVID-19, China published articles in a relatively early stage (Figure 2C), followed by Germany and the United States. And the publications from India, Canada, and Switzerland were issued at a relatively late in time course. Of note, multinational co-authored publications from China, England, Canada, France, and the Netherlands had higher average citations (Figure 2D).

### Institutions and journals publishing articles on COVID-19 and immune response

**Figure 3:**
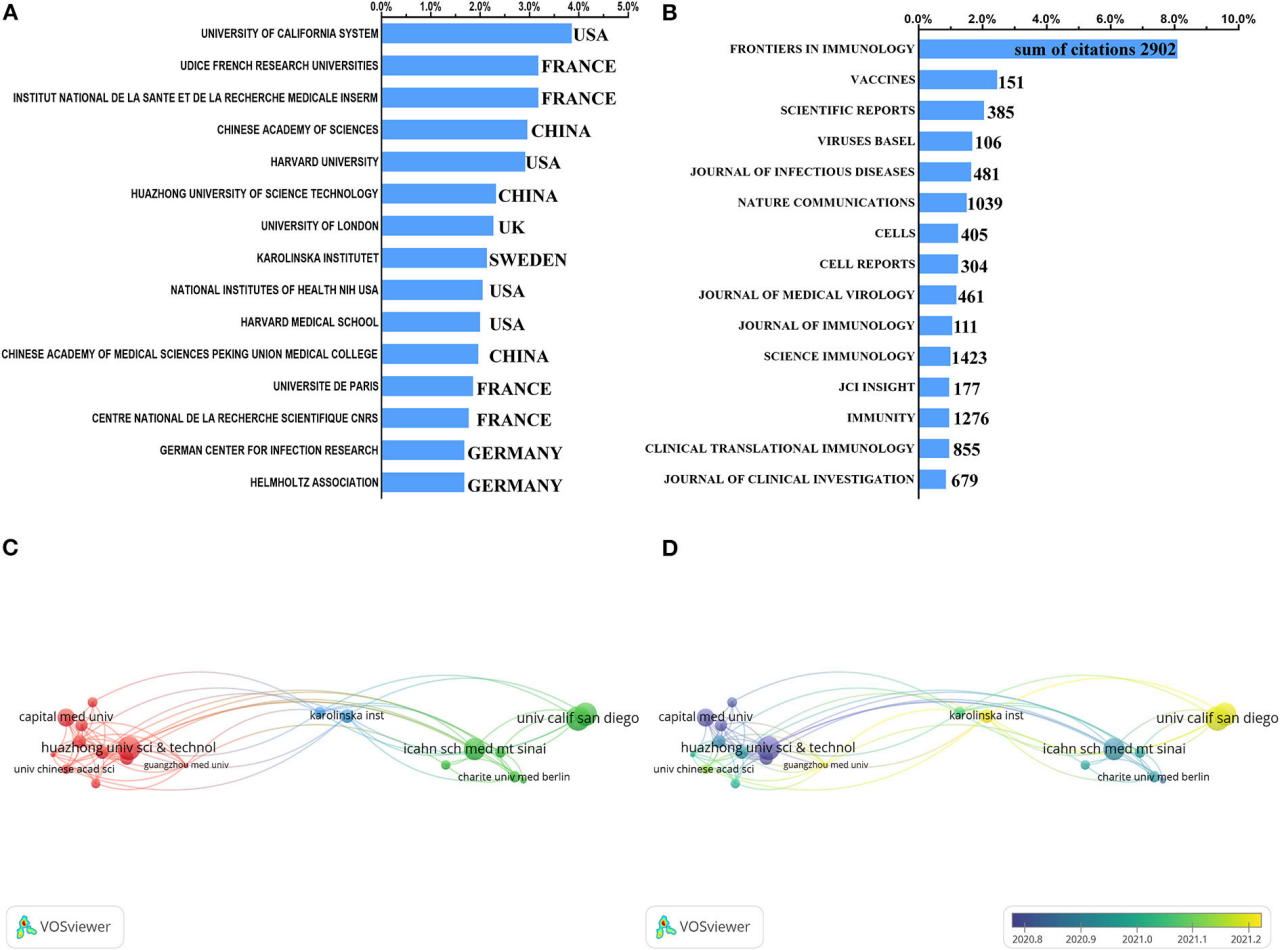
Institutions and journals publishing articles on COVID-19 and immune response. **(A)** Top 15 institutions with the most publications on immunization and COVID-19. **(B)** The ranking list of global journals publishing the most articles on immunization and COVID-19. The X-axis represented the percentage of the published number of each institution or journal to the total number of publishments. **(C)** The co-authorship network of top 20 institutions, and per institution had more than 20 publications with international cooperation. **(D)** The top 20 institutions were shown in chronological order of publications.

The top 15 most influential institutions and journals in the field of COVID-19 and immune response were shown in Figures 3A,B. Among the top 15 organizations that published the most articles on immune response and COVID-19, four of them were French institutions, four institutions belonged to the United States, and three were from China. Notably, 10% of the total articles were published by these four French institutions, although the French total number of publications ranked sixth in the top 15 countries. In addition, the University of California System had published 3.86% of the total number of publications, ranking first among the 15 institutions (Figure 3A). The minimum number of co-authorship across organizations was set to 20 and analyzed by VOSviewer, whose results were given in Figures 3C,D. The size of the circle was represented the citations of publications by every organization and the lines between circles were represented the cooperated link of institutions. Karolinska University Hospital and Karolinska Institute at the center of the map had the closer cooperation with other institutions. University of California San Diego, Huazhong University of Science and Technology, La Jolla Institute for Immunology, Icahn School of Medicine at Mount Sinai, and Capital Medical University were the top five institutions with the most citations of publications. Obviously, Chinese institutions were inclined to domestic cooperation lacking international communications (Figure 3C). In chronological order, the latest research progress was severally published by Karolinska Institute, Guangzhou Medical University, La Jolla Institute for Immunology, University of California San Diego (Figure 3D).

As for the top-ranking journals issuing publications on this topic (Figure 3B), the number of articles published on *Frontiers in Immunology* (IF: 7.561, 2020) was significantly higher than that of the other journals, with 178 records. *Vaccines* (IF: 4.422, 2020) published 54 articles in this area, which ranked second. The number of publications on *Scientific Reports* (IF: 4.379, 2020) tied for third place (45 articles). Additionally, the *Journal of Medical Virology* (IF: 2.327, 2020) also had 461 citations for 26 articles on this topic. It is notable that articles published on *Science Immunology* (22, 1,423 citations), *Immunity* (21, 1,276 citations), and *Nature Communications* (33, 1,039 citations) were ~1/8 to 1/4 of those published on *Frontiers in Immunology*, while their citation frequency per article was significantly higher than that of the *Frontiers in Immunology*.

### Authors publishing articles on COVID-19 and immune response

The top five authors with the most publications are shown in Table 1, exploring the contributions of global authors to the field of COVID-19 and immune response. The five productive authors who published the most articles were from the United States and Sweden, and the United States possessed 4 by them. The three American authors, Alessandro Sette, Daniela Weiskopf, and Alba Grifoni, were from the same institution, La Jolla Institute for Immunology, which ranked top three regarding the number of publications, citations, and H-index (as presented in Table 1). Marcus Buggert and Zhang Yun were ranked fourth and fifth with 12 (821 times citations) and 3 articles (599 times citations), respectively.

**Table 1:** Top 5 authors publishing articles on COVID-19 and immune response.

**Author**	**Country**	**Institution**	**Number of publications**	**Citations**
Alessandro Sette	USA	La Jolla Institute for Immunology	31	3,633
Daniela Weiskopf	USA	La Jolla Institute for Immunology	25	3,106
Alba Grifoni	USA	La Jolla Institute for Immunology	24	3,389
Marcus Buggert	Sweden	Karolinska University Hospital	12	821
Yun Zhang	China	J. Craig Venter Institute, La Jolla	3	599

Meanwhile, we listed the top 10 most influential articles about COVID-19 and immune response (Table 2). The article entitled “Dysregulation of Immune Response in Patients with Coronavirus 2019 (COVID-19) in Wuhan, China” ranked first regarding the citation frequency (2,450 times), in which Tian et al. conducted a case-control study of COVID-19 patients hospitalized in Wuhan (23). “Targets of T Cell Responses to SARS-CoV-2 Coronavirus in Humans with COVID-19 Disease and Unexposed Individuals” (24) published on *Cell* by Sette A had the second citation (1,530 times). The third most influential article was a retrospective review performed by Chen YW from China, in which they revealed the phenomenon of the decrease, functional exhaustion of T cells in COVID-19 patients and a negative correlation between T cell count and patient survival (1,057 times of citations) (25). It was noteworthy that 4 of the 10 most influential articles adopted advanced sequencing technologies (24, 28, 30, 32).

**Table 2:** Lists of the top 10 most-cited articles.

**Title**	**Corresponding authors**	**Journals**	**Publication date**	**Total citations**
Dysregulation of immune response in patients with coronavirus 2019 (COVID-19) in Wuhan, China (23)	Tian DS	Clinical Infectious Diseases	2020.08	2,450
Targets of T cell responses to SARS-CoV-2 coronavirus in humans with COVID-19 disease and unexposed individuals (24)	Sette A	Cell	2020.06	1,530
Reduction and functional exhaustion of T cells in patients with coronavirus disease 2019 (COVID-19) (25)	Chen YW	Frontiers in Immunology	2020.05	1,057
Pathological inflammation in patients with COVID-19: a key role for monocytes and macrophages (26)	Martin JC	Nature Reviews Immunology	2020.06	947
The role of cytokines including interleukin-6 in COVID-19 induced pneumonia and macrophage activation syndrome-like disease (27)	Bridgewood C	Autoimmunity Reviews	2020.06	776
SARS-CoV-2-specific T cell immunity in cases of COVID-19 and SARS, and uninfected controls (28)	Bertoletti A	Nature	2020.08	748
Targeting potential drivers of COVID-19: neutrophil extracellular traps (29)	Egeblad M	Journal of Experimental Medicine	2020.06	690
Robust T-Cell Immunity in Convalescent Individuals with Asymptomatic or Mild COVID-19 (30)	Buggert M	Cell	2020.08	615
Neutrophil extracellular traps in COVID-19 (31)	Knight JS	JCI Insight	2020.06	592
Potent neutralizing antibodies against SARS-CoV-2 identified by high-throughput single-cell sequencing of convalescent patients' B cells (32)	Xie XS	Cell	2020.07	553

### Research hotspots on COVID-19 and immune response

**Figure 4:**
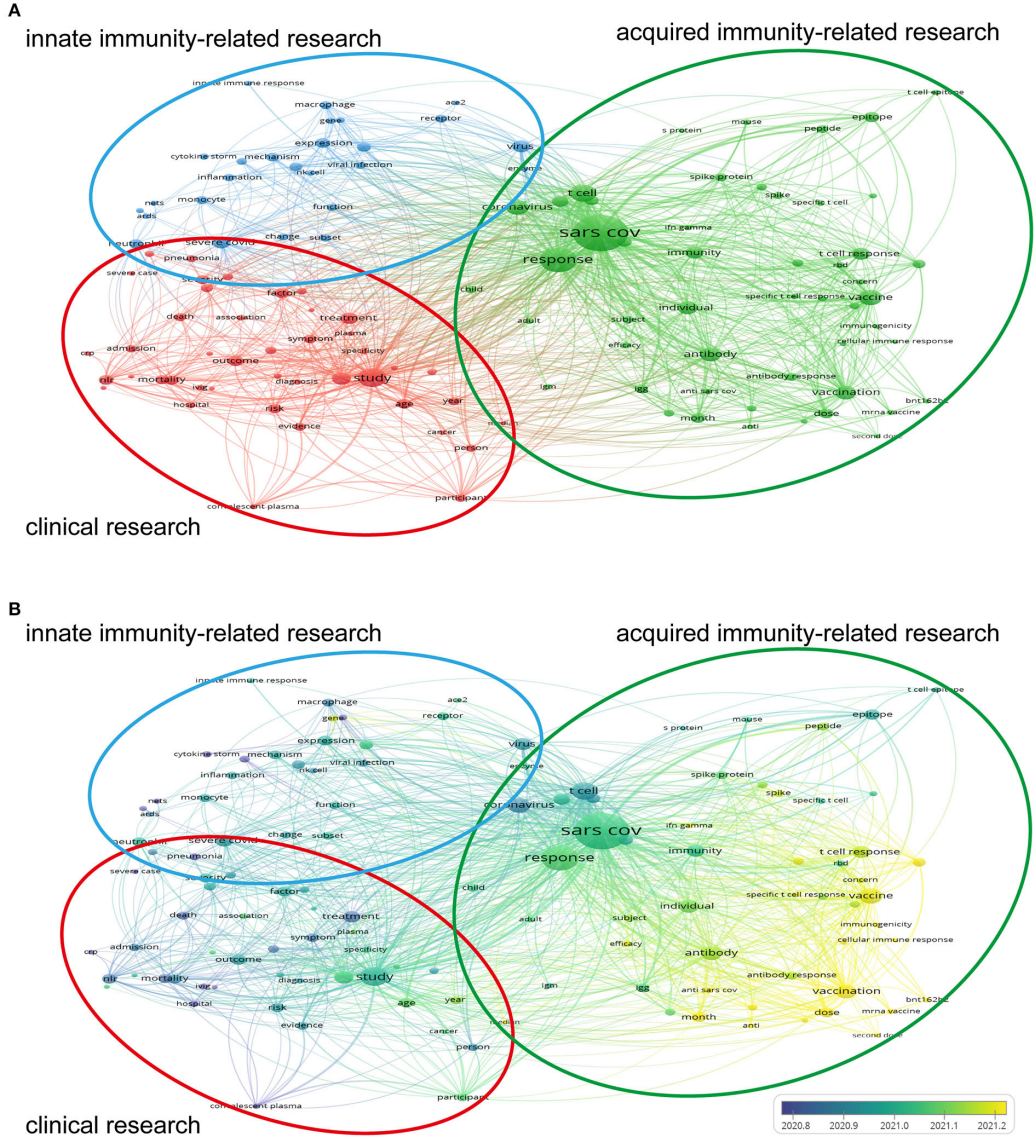
Cluster analysis diagram of research hotspots. Keywords that appeared at least 85 times in titles and abstracts were analyzed by VOSviewer software. The larger the circle of the keyword, the more frequently it appeared. The co-occurrence times of two keywords determined the distance between them. **(A)** The keywords were classified into three clusters: clinical research (red), acquired immunity-related research (green), and innate immunity-related research (blue). **(B)** Keywords were colored in chronological order. Purple keywords were the early-emerging ones, whereas keywords in yellow appeared more recently.

To understand the main research focus of the 2,200 publications in detail, we applied the VOSviewer Software to analyze the keywords extracted from the titles and abstracts, which were defined as words appearing at least 85 times. A total of 120 keywords met the requirements and were subsequently categorized into three clusters: clinical research, innate immunity-related research, and acquired immunity-related research (Figure 4A). Within the cluster of “clinical research”, the following keywords frequently appeared: study (1,712 times), group (806 times), treatment (621 times), outcome (550 times), and NLR (neutrophil-lymphocyte ratio, 498 times). In the subset of “innate immunity-related research”, virus (716 times), severe COVID (687 times), expression (457 times), activation (434 times), and neutrophil (408 times) represented the primary keywords within the publications. The relevant keywords in a cluster of “acquired immunity-related research” were also listed: SARS COV (6,431 times), response (2,947 times), T cell (1,467 times), vaccine (1,173 times), and coronavirus (1,102 times).

As summarized in Figure 4B, the VOSviewer assigned keywords with disparate colors in line with the average appearing time, for which purple keywords were the early-emerging ones, whereas keywords in yellow appeared more recently. In terms of the whole cluster analysis graph, “convalescent plasma,” “severe case,” “march,” “pneumonia,” and “cytokine storm” represented the well-studied topics in the early stage of research on immune response and COVID-19, whereas “humoral response,” “variant,” “vaccination,” “second dose,” and “BNT162b2” (the mRNA vaccine of Pfizer–BioNTech) had become the recent foci. Notably, these five newest words were all presented in the cluster of “acquired immunity-related research”, and these early-emerging keywords were principally fallen under the “clinical research” cluster. For each cluster individually, “median” was the lasted keyword in this subset of “clinical research”, with 86 times of co-occurrence. Similarly, “year” (316 co-occurrences), “plasma” (113 co-occurrences), “age” (480 co-occurrences), and “association” (175 co-occurrences) were the newest terms in this cluster. The keywords “gene” (interferon, 214 co-occurrences), “ACE2” (angiotensin-converting enzyme 2, 113 co-occurrences), “activation” (434 co-occurrences), “enzyme” (118 co-occurrences), and “receptor” (321 co-occurrences) were the recently emerging words in the cluster of “innate immunity-related research”. Unlike the other two clusters, the green cluster of “acquired immunity-related research” gathered most of the latest keywords. And “coronavirus” (1,102 co-occurrences), “development” (444 co-occurrences), “T cell” (1,467 co-occurrences), “S protein” (111 co-occurrences), and “epitope” (518 co-occurrences) were the earlier-appeared keywords in this cluster. Additionally, we applied CiteSpace in performing a secondary clustering analysis on keywords. The 10 largest clusters were shown in the Supplementary Figure 1a. The sequence number was inversely proportional to the size of the cluster, i.e., #0 antibody response was the largest cluster. The name of each cluster was default by the software based on the keywords' characteristics. Supplementary Figure 1b shown the keywords with high frequency in every cluster and their connections. And, the top 10 representative keywords in each cluster were shown in Supplementary Table 1 in line with their co-occurrence frequency. The representative keywords of the #0 cluster were antibody, cell, antibody response, influenza, therapy, immunogenicity, cancer, cellular, immunity, CD4^+^ T cell, and safety.

### Analysis of references in publications on COVID-19 and immune response

**Figure 5:**
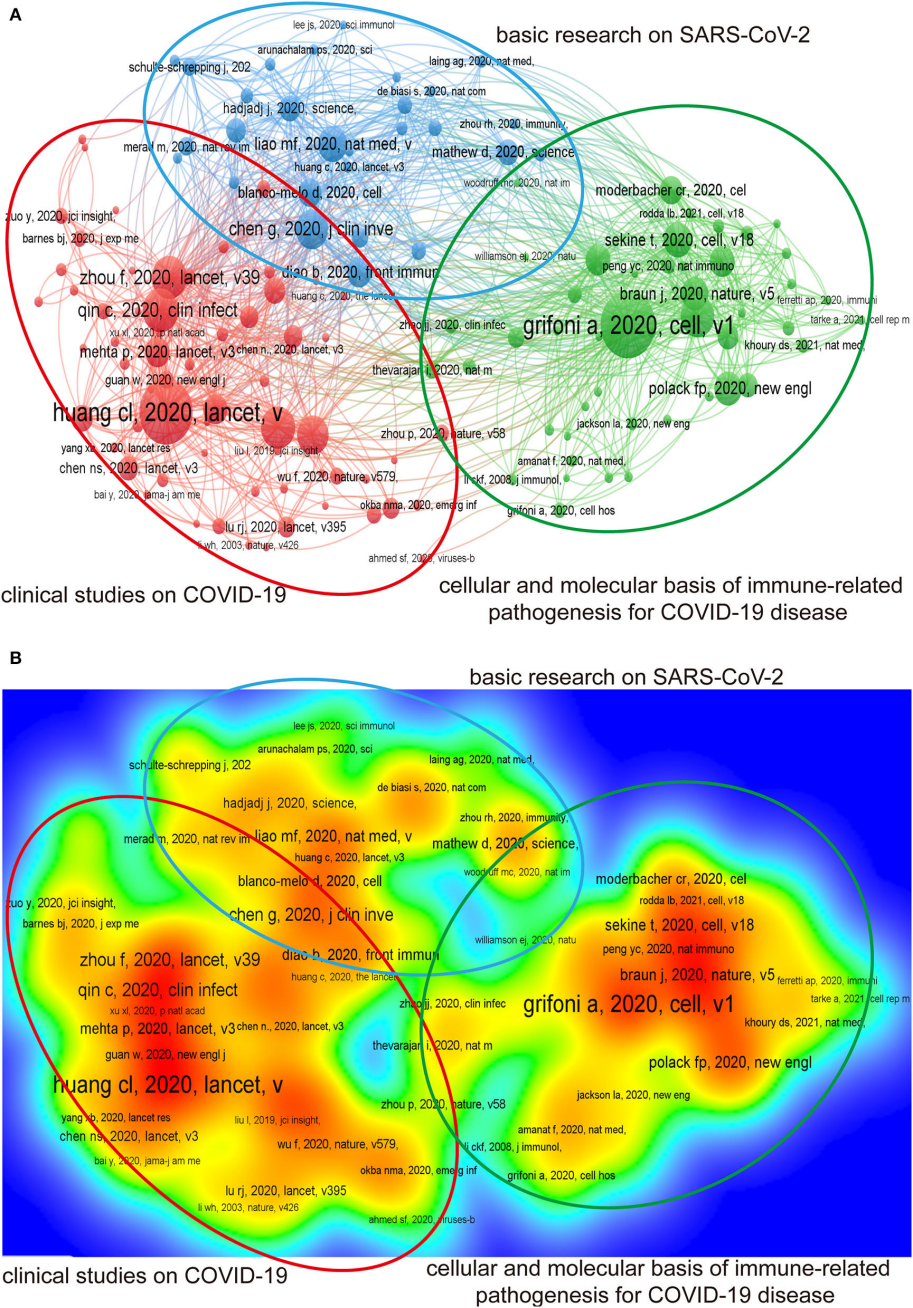
Cluster analysis plot of references for co-citation. Top 139 articles cited at least 50 times were analyzed by VOSviewer. **(A)** Each circle in the figure represented a reference of co-citation, and the circle size was proportional to the number of citations. All references were divided into 3 clusters according to their contents: clinical studies on COVID-19 (red), basic research on SARS-CoV-2 (blue), and the cellular and molecular basis of immune-related pathogenesis for COVID-19 disease (green). **(B)** The thermodynamic chart of references. All references of co-citation were colored difference according to their citations. References with the highest number of citations were marked in red.

A total of 139 references cited at least 50 times were retrieved and eligible for analyzing co-citation, which was further classified into three clusters (Figure 5). The red cluster contained 64 articles that involved clinical studies on COVID-19. The blue subset included 28 records of basic research on SARS-CoV-2. In addition, the third cluster in green documented 47 articles focusing on the cellular and molecular basis of immune-related pathogenesis for COVID-19 (Figure 5A).

As shown in the density visualization of Figure 5B, Alba Grifoni's work entitled “Targets of T Cell Responses to SARS-CoV-2 Coronavirus in Humans with COVID-19 Disease and Unexposed Individuals” was identified as the first ranked reference (415 times of co-citation), which belonged to the cluster associated with the cellular and molecular basis of immune-related pathogenesis for COVID-19 (24). The other four studies of the top five co-cited references were all clustered in the group annotated as “clinical studies on COVID-19”. And the article “Clinical features of patients infected with 2019 novel coronavirus in Wuhan, China” conducted by Huang CL et al. was the second most influential by 406 co-citations up to the present (33). In addition, the other three studies were performed by Qin C (270 times of co-citation) (23), Zhou F (264 times of co-citation) (34), and Hoffmann M (259 times of co-citation) (35), respectively. Strikingly, three of the top five influential references regarding times of co-citation were published by scholars in China.

## Discussion

### Research trends of COVID-19 and immune response

The United States, China, and Germany were the top three countries for the total number of publications, citations, and H-index among all countries/regions (Figure 1A). China firstly reported the genetic sequence of the SARS-CoV-2 and the clinical manifestations of COVID-19, which had unprecedented impacts on the diagnosis and treatment of COVID-19. Nevertheless, even though China had first-hand clinical data earlier, the quality and quantity of publications on COVID-19 and immunity were gradually caught up by the United States.

As depicted in Figure 5, the majority of Chinese publications fell into the cluster of clinical studies on COVID-19, whereas merely fractional of cited Chinese articles associated with basic research, as well as immune-related pathogenesis on COVID-19. However, the rapid global spread of SARS-CoV-2 prompted the publication of the United States and other countries. Meanwhile, the outbreak in China was quickly brought under control due to the vigorous efforts implemented by the local government. Given that, the decline in the number of cases also partially explained why China could not maintain its advantage in clinical research. The basic research conditions in the United States seem superior to other countries, as the support by advanced experimental equipment, sufficient scientific funding, and high-end professionals. Notably, the application of some cutting-edge sequencing technologies for deciphering immune heterogeneity was more sophisticated in the United States. Therefore, the advantages mentioned above have explained why the United States could catch up with China's outputs in a relatively shorter time and make the most indispensable contribution in this field. Correspondingly, it suggests the urgent need for China to promote the quality of the publications by carrying out more in-depth basic research on COVID-19-associated immune response.

The international cooperation of the top 15 countries/regions was shown in Figure 2C, for which the distances between each country/region determined the closeness of the cooperation, and the size of the circle was positively related to the number of documents. Overall, the United States and Switzerland cooperated closely with other countries, as evidenced by their relatively central position in the network. However, China was in a relatively marginal position, indicating a lack of international cooperation. To a certain extent, frequent international cooperation can effectively elevate the quality and impact of publications and boost the academic influence on scholars and institutions in this field. Within the context of the global pandemic and rapid mutations of the virus, China and other countries should potentiate international cooperation and academic exchanges to conduct more in-depth and comprehensive research on COVID-19 and immune response in the future.

In terms of institutions publishing research on COVID-19 and immune response, the University of California System in the USA ranked first, followed by Udice French Research Universities and the National Institute of Health and Medical Research in France. Institutions from France, the United States, and China dominated this research field, as evidenced by 4 of the top 15 institutions being from France, 4 of them from the United States, and 3 from China. As for the journals, *Frontiers in Immunology* published the most articles with the highest total citations on COVID-19 and immune response compared to the others since this journal was open access with an irregular publishing period. Meanwhile, *Frontiers in Immunology* set up various sections related to COVID-19 by inviting professional researchers worldwide, leading to the publication of more influential and advanced studies. Notably, other journals with huge academic impact included *Science Immunology, Immunity, Nature Communications*, and *Journal of Medical Virology* also published several articles on this topic. Although few of their publications had relatively higher citation frequency per article, indicating their profound academic influence, we forecast that the latest research progress in this field may still appear in *Frontiers in Immunology* and those journals, as supported by their fame and impact in this area.

As for the most prolific and influential authors in the field of immune response and COVID-19, the top three authors, Alessandro Sette, Daniela Weiskopf, and Alba Grifoni, were all from the team of the La Jolla Institute for Immunology in the United States. They have 21 co-authored articles. And research conducted by this team comprehensively covered the role of T cells in SARS-CoV-2 infection, clinical symptoms of COVID-19, and immunization vaccine. However, the co-authorship network in Figure 3C showed that this productive group lacked international interaction. Chinese institutions that also lack international interaction prefer domestic cooperation, which is not conducive to the country's research progress in this field. Therefore, the collaboration across scholars should be promoted to yield more influential studies, facilitating a better understanding of the immune response elicited by SARS-CoV-2 infection.

### Research focus in the field of COVID-19 and immune response

These top 10 publications with the highest citation frequency mainly focused on the immunological manifestations and early diagnosis of COVID-19 cases, as well as the immunopathogenesis of COVID-19 (Table 2). Among the top 10 most influential articles, “Dysregulation of Immune Response in Patients with Coronavirus 2019 (COVID-19) in Wuhan, China”, had been cited 2,450 times and published in *Clinical Infectious Diseases* by Tian et al. (23). In this observational study, the author revealed the laboratory testing values of 452 COVID-19 patients for the prediction of severe cases. They found that SARS-CoV-2 might primarily attack T lymphocytes, resulting in an elevation in the neutrophil-lymphocyte ratio (NLR). It implied that monitoring of T lymphocyte subsets and NLR could improve the early recognition and diagnosis of COVID-19. Another study published by Sette A et al. on *Cell* (24) investigated the characteristics of CD4^+^ and CD8^+^ T-cell immune responses among confirmed COVID-19 cases. They noticed that CD4^+^ T cells were activated in response to spike protein 100%, and the intensity of response to spike protein was correlated with the titers of anti-SARS-CoV-2 IgG and IgA. Their ORF (open reading frame) mapping of T cell specificities might provide valuable targets for most vaccine development. In addition, the team determined the SARS-CoV-2-reactive CD4^+^ T cells in ~40**–**60% of unexposed healthy controls, implicating the common coronavirus had the identical cross-reactive T-cell recognition with SARS-CoV-2. Within similar items, Bertoletti et al. reported their investigations concerning cross-reactive T-cell recognition of SARS-CoV-2 in *Nature* (28). In addition to comparing the T cell immunity between COVID-19 cases and unexposed healthy controls, Bertoletti et al. included SARS cases in their study. After focusing on T cell response against nucleocapsid (N) proteins, non-structural protein 7 (NSP7), and NSP13 of convalesced COVID-19 patients, they found CD4^+^ and CD8^+^ T cells could recognize multiple regions of N protein. Moreover, these SARS-CoV-2-specific T cells in unexposed individuals targeted NSP7, NSP13, and N protein in a distinct pattern. Besides, T cells from 23 convalesced patients had strong cross-reactivity to the N protein of SARS-CoV-2 after infection with SARS, which displayed a long-lasting memory function. Collectively, these studies on the response of T cells to SARS-CoV-2 have partially explained the immunopathogenesis in different populations, which might be of great significance for the control of the COVID-19 pandemic.

The VOSviewer extracted the in-text keywords with the most frequent co-occurrences and categorized them into 3 clusters (Figure 4). The top 5 keywords regarding co-occurrence in each cluster basically represent the main research focus. Correspondingly, we annotated these three clusters as “clinical research,” “acquired immunity-related research,” and “innate immunity-related research,” respectively. Most of the latest research came from the green cluster in “adaptive immunity related-research”. Therefore, it is not hard to speculate that the development and evaluation of vaccines should be a recent hotspot.

Of note, studies on T-cell immunity accounted for more than 50% of all publications on COVID-19 and immune response. Many retrospective analyses indicate that T cells might play a pivotal role in anti-coronavirus immunity. Diao et al. showed that COVID-19 patients had undergone a substantial decline in CD4^+^ and CD8^+^ T cell counts, accompanied by evident functional exhaustion (25). Likely, the study by Li S et al. revealed that the inflammatory state and functional defect of CD4^+^ T cells in severe cases could be the key player in the pathogenesis and recovery of COVID-19 (36). Another study showed that humoral immune response level and T cell immune memory were positively correlated with the severity of COVID-19 (35). Additionally, the prolonged positivity of SARS-CoV-2 was associated with the suppressed differentiation of CD8^+^ Teff (effector T cell) and Tem (effector memory T cell) (37). Given their crucial impacts on the development of COVID-19, many researchers have begun to propose effective antiviral treatments by targeting T-cell immunity. For example, thymosin alpha-1 reportedly reduced the mortality of critical patients confirmed with SARS-CoV-2 infection *via* promoting thymic output, reversing T-cell exhaustion, and improving immune function (38). The treatments involving immune checkpoint inhibitors were demonstrated to enhance T-cell immunity but not aggravate the systemic inflammation during the progression of COVID-19 (39). Furthermore, several studies found that COVID-19 patients had SARS-CoV-2-specific T-cell immune memory after clinical recovery, which could last for at least 6 months (37, 40). These findings on T-cell immunity provide optimal targets and promising prospects for the research and development of vaccines and specific remedies against SARS-CoV-2 infection.

### The hotspot in future

The clustering analyses of keywords reveal that vaccine development and efficacy evaluation will soon become a hot research topic in the future. Accordingly, there are 232 vaccine candidates in disparate stages of development worldwide, of which nine have been approved and put into clinical use in many countries (41). Currently, mRNA, adenovirus, and inactivated vaccines have accounted for more than 95% of global vaccines (42). In vaccinated and unvaccinated populations, vaccine efficacy is assessed by three primary endpoints, including the proportion of recorded infections, symptomatic infections, and severely hospitalized cases (43). Meanwhile, the titers of RBD-specific IgG, neutralizing antibodies, and T cytokine levels (especially the IFN-γ levels) are adopted as the indicators to determine whether the vaccine induced an effective humoral and cellular immune response in the host (44). The effectiveness of COVID-19 vaccines has been evaluated in healthy adults (45), immunocompromised patients (46), adolescents and children (47, 48), pregnant women (49), and cancer patients (50). The evaluation results showed that vaccination has distinct degrees of immune protection in all populations. Specifically, for the elderly and immunocompromised individuals, a different vaccine formulation or booster dose is recommended to reinforce the immune response (50, 51). Despite the effectiveness of vaccination and booster vaccination in preventing symptomatic infections and severe cases, we still encounter substantial challenges regarding breakthrough infections caused by variants and uneven distribution of global vaccination quotas, which require further investigation in the future.

## Limitations

Several limitations should be considered when interpreting our findings. Firstly, a fraction of non-English publications was not considered due to our inclusion criteria. Omission of articles published in non-English language might inevitably render some inaccuracy. Secondly, we solely extracted records from the WOS, rather than databases such as Scopus and PubMed. Finally, since articles on COVID-19 and immunity were mainly published in the past 2 years and updated rapidly, we were unable to fit the growth curve for each country/region to precisely predict the quantity of publication in the upcoming years.

## Conclusion

Taken together, the current study has summarized and elucidated the global publication trend of research on COVID-19 and immune response. Among all eligible records, the United States has the most publications with the highest H-index and citation frequency. China ranks second in the publication number, total citations, and H-index. Based on our bibliometric analysis, China still needs to strengthen its international cooperation and basic research. Scientific journals, including *Frontiers in Immunology, Science Immunology, Immunity, Nature Communications*, and *Journal of Medical Virology*, will publish more influential work and elicit the latest progress in COVID-19 immunity. Alessandro Sette represents the most influential scholars on this topic. By analyzing keywords, the development and evaluation of vaccines become a novel research hotspot in relevant area.

## Data availability statement

The datasets presented in this study can be found in online repositories. The names of the repository/repositories and accession number(s) can be found in the article/Supplementary material.

## Author contributions

X-mS, Y-mY, and R-qY conceived the analysis. YX and P-yZ extracted all data. Z-bT undertook and refined the inclusion process. YX, R-qY, and Y-mY co-wrote the article. R-qY, YX, P-yZ, L-yZ, and H-tZ undertook the bibliometric analyses. All authors contributed to and revised the final manuscript.

## Funding

This work was supported by grants from the National Natural Science Foundation of China (Nos. 81571941, 82172144, and 82130062), the Funds for Young Reserve Talents of Hubei Province of China (No. HBRC20200411), and National Tutorial System Training Program of Suzhou (2020-12).

## Conflict of interest

The authors declare that the research was conducted in the absence of any commercial or financial relationships that could be construed as a potential conflict of interest.

## Publisher's note

All claims expressed in this article are solely those of the authors and do not necessarily represent those of their affiliated organizations, or those of the publisher, the editors and the reviewers. Any product that may be evaluated in this article, or claim that may be made by its manufacturer, is not guaranteed or endorsed by the publisher.

## Supplementary material

Supplementary Figure 1 The secondary cluster analysis of keywords by CiteSpace. **(a)** Top 10 cluster with the most keywords according to the LRR algorithm. The sequence number was inversely proportional to the size of the cluster, i.e., #0 is the largest cluster. The name of each cluster was default by the software based on the keywords' characteristics. **(b)** The keywords with high frequency in every cluster and their connections. The representative keywords in each cluster were shown in Supplementary Table 1.

Supplementary Table 1 Top 10 clusters by size and their representative keywords in CiteSpace based on the log-likelihood ratio (LLR) algorithm.
