# Supplementary figures and images for: Publication trends of research on COVID-19 and host immune response: A bibliometric analysis
Source: Front Public Health. 2022 Aug 8;10:939053. doi: 10.3389/fpubh.2022.939053 (PMC9394856; doi:10.3389/fpubh.2022.939053)

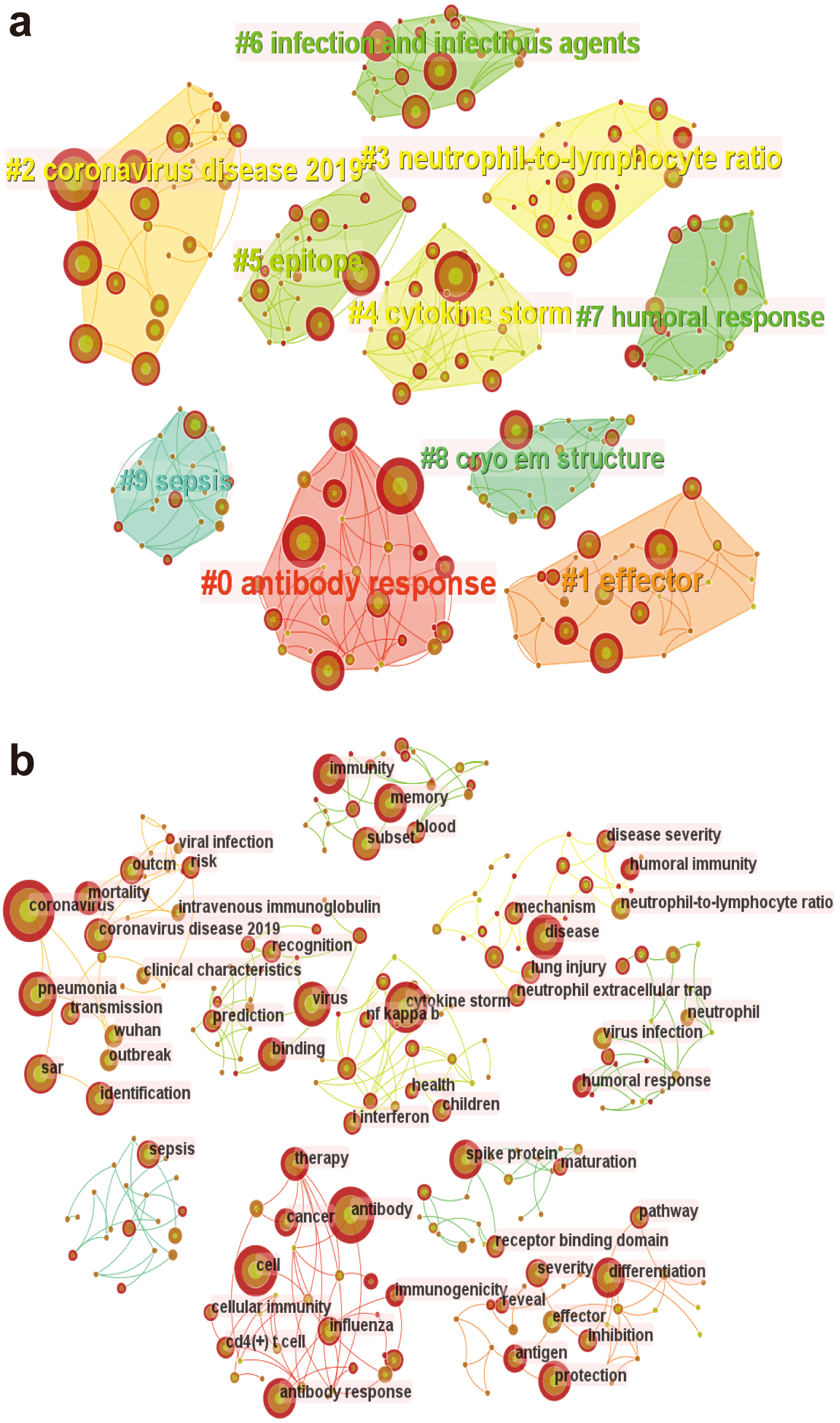

Supplement: Supplementary Figure 1 — The secondary cluster analysis of keywords by CiteSpace. (a) Top 10 cluster with the most keywords according to the LRR algorithm. The sequence number was inversely proportional to the size of the cluster, i.e., #0 is the largest cluster. The name of each cluster was default by the software based on the keywords' characteristics. (b) The keywords with high frequency in every cluster and their connections. The representative keywords in each cluster were shown in Supplementary Table 1. [file Image_1.tif]
