# Supplementary material for: Publication trends of research on COVID-19 and host immune response: A bibliometric analysis
Source: Front Public Health. 2022 Aug 8;10:939053. doi: 10.3389/fpubh.2022.939053 (PMC9394856; doi:10.3389/fpubh.2022.939053)
Supplement: Supplementary Table 1 — Top 10 clusters by size and their representative keywords in CiteSpace based on the log-likelihood ratio (LLR) algorithm. [file Table_1.docx]

**Supplementary table 1. Top 10 clusters by size and their representative keywords in CiteSpace based on the log-likelihood ratio (LLR) algorithm.**

| **Cluster** | | **Ten representative keywords** | |
| --- | --- | --- | --- |
| #0 antibody response | antibody, cell, antibody response, influenza, therapy, immunogenicity, cancer, cellular, immunity, CD4^+^ T cell, safety | | |
| #1 effector | protection, differentiation, severity, antigen, effector, inhibition  pathway, reveal, interferon gamma, chemokine | | |
| #2 coronavirus disease 2019 | coronavirus, pneumonia, SARS, coronavirus disease 2019, mortality, identification,  Wuhan, outcome, intravenous immunoglobulin, transmission | | |
| #3 neutrophil-to-lymphocyte ratio | disease, mechanism, neutrophil extracellular trap, humoral immunity,  neutrophil-to-lymphocyte ratio, disease severity, lung injury, flow cytometry,  oxidative stress, risk factor | | |
| #4 cytokine storm | cytokine storm, I interferon, NF-kappa-B, children, health, acute lung injury,  apoptosis, gene, macrophage activation syndrome, extracellular trap | | |
| #5 epitope | virus, binding, prediction, recognition, mutation, diversity  epitope, molecule, signature, human coronavirus | | |
| #6 infection and infectious agents | immunity, memory, subset, blood, memory B cell, infectious disease,  memory T cell, dynamics, cytokine release syndrome, CD8^+^ T cell | | |
| #7 humoral response | virus infection, humoral response, neutrophil, IgG, monoclonal antibody,  nucleocapsid protein, immune response, antibody dependent enhancement,  cell response, type I interferon | |  |
| #8 cryo-EM (Cryo-electron microscopy) structure | spike protein, receptor binding domain, maturation, functional receptor,  neutralization, COV, angiotensin converting enzyme 2, suppressor cell,  clinical trial, HIV-1 | |  |
| #9 sepsis | sepsis, IL-6, impact, adult, vitamin D, SARS-CoV-2, case report,  C reactive protein, IVIg, acute kidney injury | |  |
